# Non-invasive brain stimulation on functional capacity in prefrail older adults: a protocol for a randomised clinical trial

**DOI:** 10.3389/fnagi.2026.1891571

**Published:** 2026-08-12

**Authors:** Pilar Serra-Añó, Elena Muñoz-Gómez, Sara Mollà-Casanova, Noemí Moreno-Segura, Héctor González-Pons, Marta Inglés

**Affiliations:** 1 Research Unit in Clinical Biomechanics (UBIC), Department of Physiotherapy, Faculty of Physiotherapy, Universitat de València, Valencia, Spain; 2 Neuro-UBIC, INCLIVA Biomedical Research Institute, Valencia, Spain

**Keywords:** functional capacity, non-invasive neuromodulation, prefrail, tDCS, virtual running

## Abstract

**Introduction:**

Therapeutic physical exercise (TPE) is considered the cornerstone intervention to prevent or reverse frailty; however, prefrail individuals often face challenges achieving the exercise intensity required to induce multisystem adaptations. Non-invasive neuromodulation techniques—including virtual running–based visual illusion (VR) and, transcranial direct current stimulation (tDCS),—have shown promising effects on neuroplasticity, motor performance and executive function. The primary objective of this study is to compare the effect of adding two neuromodulation strategies to a multicomponent TPE programme on functional capacity and quality of life in prefrail older adults.

**Methods and analysis:**

This four-arm, triple-blinded randomized controlled trial will include adults aged ≥ 65 years classified as prefrail according to Fried criteria. Participants will be randomly assigned to one of the following groups: VR+TPE, sham VR+TPE, tDCS+TPE or, sham tDCS+TPE. All interventions will be applied during an 8 week-programme consisting of 24 supervised sessions. The primary outcomes will be frailty status (Fried Phenotype) and walking speed. Secondary outcomes will include neuroplasticity biomarkers (plasma BDNF), heart rate variability, upper and lower limb isometric strength, quality of life (EQ 5D 5L), lower-limb muscle power, static and dynamic functionality, gait quality and dual-task performance. Assessments will be conducted at baseline, immediately after the first session, at 4 weeks, at the end of the 8 week intervention, and at 4 week follow up.

**Ethics and dissemination:**

The study has received approval from the Ethics Committee of Research in Humans of the University of Valencia (2025-FIS-4007899). All participants will provide written informed consent before enrolment. Study findings will be disseminated through peer reviewed publications, scientific conferences and participant summaries.

**Clinical trial registration:**

https://clinicaltrials.gov/study/NCT07346989?cond=NCT07346989&viewType=Card&rank=1, identifier NCT07346989.

## 1 Introduction

The ageing of the population poses a challenge to current public health systems, as people over 65 account for a significant proportion of healthcare expenditure (Sinha, 2011). In fact, as life expectancy increases, so does the prevalence of age-related diseases and disabilities (Lejoyeux et al., 2008). Therefore, the current trend is to promote healthy ageing, making it necessary to identify strategies to prevent disability (Viña and Borrás, 2024).

An example of unhealthy ageing is frailty, a geriatric syndrome that identifies people who are more vulnerable to external stressors, thereby increasing their risk of institutionalization, disability or even death (Fried et al., 2001). The negative effects of frailty are not limited to physical deterioration; they also impact the social, psychological and cognitive spheres of older people, thus reducing their quality of life (Clegg et al., 2013). Frailty is mostly characterized by impairments in the physical domain, such as muscle strength and gait (Pritchard et al., 2017). However, an emerging field of research is focused on the fact that frailty could also be associated with deterioration in brain health (Gutiérrez-Zúñiga et al., 2023), since it has been related to impairment in the brain microstructure (both grey matter and white matter) (Maltais et al., 2020; Tian et al., 2020). In this regard, the reduction of grey matter in specific areas such as the frontal and medial-temporal regions of the brain has been associated with a decrease in walking speed, one of the key criteria for frailty (Callisaya et al., 2014; Rodrigue and Raz, 2004; Rosano et al., 2012).

Furthermore, frailty is associated with a reduction in sensory and motor cortical representation, which affects crucial functions such as sensorimotor coordination, motor function in general, and gait and balance in particular (Carp et al., 2011; Goh, 2011; Koen et al., 2019). This, in turn, leads to a decrease in perceptual impulses, motor actions, and therefore, overall brain stimulation (Dinse, 2006; Mahncke et al., 2006). This causes cortical reorganization, resulting in altered motor patterns, such as motor instability, coordination deficits and decreased movement speed (Dinse, 2006; Mahncke et al., 2006). However, one of the most important characteristics of frailty, from a therapeutic point of view, is that it refers to a dynamic and reversible condition, which means that not all individuals are frail in the same way and, furthermore, a frail individual can become non-frail or robust if frailty is detected and treated, especially in the prefrailty state (Abellan van Kan et al., 2008).

Therapeutic physical exercise (TPE) is the most widely used therapeutic intervention for addressing frailty, especially multicomponent exercise. For TPE to induce multisystemic beneficial effects (including neuroplasticity) and thus improve physical and cognitive status, it must be performed at an appropriate intensity and frequency (World Health Organization, 2010). However, physical limitations and exercise-induced fatigue in frail or prefrail older adults may hinder adherence to such prescriptions (Picorelli et al., 2014). For this reason, other therapeutic strategies are being studied to enhance the results of physical exercise. In this regard, and given that the brain is a highly dynamic organ that exhibits adaptive and maladaptive plasticity (Flor et al., 1995), brain stimulation could be an optimal target for innovative interventions that facilitate ‘executive control of movement,’ focusing on the activation of cortical areas responsible for movement representation, such as motor and premotor areas (Shamsi et al., 2022; Thieme et al., 2018).

Some of these alternatives are mirror neuron activation therapies (MNAT) (motor imagery, action observation, and mirror therapy and/or visual illusion), which have been shown to activate the cortical and subcortical neural networks where mirror neurons are located, enhancing adaptive neuroplasticity mechanisms (Kim and Lee, 2021). In this regard, several studies have demonstrated the beneficial effects of this type of therapeutic intervention on various motor functions in people with diseases such as Parkinson’s (Kwok et al., 2022), after traumatic brain injury (Maggio et al., 2020), stroke (Gandhi et al., 2020; Peng et al., 2019) or spinal cord injury (Moseley, 2007). In the latter population group, our research team has verified how a virtual walking protocol combined with a TPE programme improves their functional capacity and perception of pain. Furthermore, we have recently published a systematic review and meta-analysis showing how MNAT improves functional capacity in older people, although the results when added to TPE are inconclusive, which opens the door to study combined interventions (MNAT + TPE) in frail older people (Mollà-Casanova et al., 2024).

In this context, we recently demonstrated that a visual illusion protocol (i.e., virtual running (VR) combined with TPE improves voluntary movements (i.e., aerobic capacity, lower limb strength and reaction time) in frail and prefrail individuals (Mollà-Casanova et al., 2023). This activation of mirror neurons arises from the activity within the cortical action-perception network that is triggered by the visual stimulus, which engage neuroplastic mechanisms. However, there are different ways to enhance neuroplasticity so, it is highly relevant to explore different therapeutic alternatives in this population, such as transcranial direct current stimulation (tDCS), and to assess their impact on functional variables and to neuroplasticity biomarkers.

Specifically, tDCS consists of applying a weak direct current to the desired cortical area, to modulate brain excitability by shifting the resting membrane potential (Nitsche et al., 2003). In older people, this therapy has been shown to improve aspects of executive function related to movement (Manor et al., 2016; Zhou et al., 2015), but its effectiveness on the functional capacity of frail individuals has not been studied.

Although VR and tDCS both aim to enhance neuroplasticity, they are expected to act through partially distinct neural pathways. VR-based visual illusion primarily engages the action-observation network and mirror neuron system, involving premotor cortex, inferior parietal cortex and sensorimotor integration networks (Kim and Lee, 2021). Conversely, anodal tDCS induces subthreshold modulation of neuronal membrane potentials, facilitating Hebbian plasticity, NMDA-receptor activity and BDNF-dependent synaptic strengthening (Fritsch et al., 2010). Consequently, these interventions may produce distinct neurophysiological adaptations and functional outcomes.

Although several studies have demonstrated additive benefits of combining exercise with neuromodulation techniques in various, these approaches have generally been investigated independently and focused primarily on cognitive aspects, rather than functional. To our knowledge, no randomized controlled trial has directly compared the effect of VR and tDCS when added to a multicomponent exercise programme in parameters related to functional capacity and quality of life in prefrail individuals.

Therefore, this study hypothesizes that adding any of these two therapies (VR and tDCS) to a multicomponent TPE intervention programme will result in additional improvements in parameters related to functional capacity and quality of life in prefrail individuals. This will contribute to preventing the progression toward frailty and, therefore, reducing the social and healthcare costs associated with this condition.

**Objectives:** The general objective of this study is to evaluate the effectiveness of non-invasive neuromodulation combined with a multicomponent TPE programme on variables related to functional capacity and quality of life in prefrail older adults. As specific objectives, the current study seeks to determine the effect of two neuromodulation strategies (i.e., tDCS and VR) combined with a multicomponent TPE programme on gait speed, general physical condition, frailty, static and dynamic functionality and gait quality, upper limb and lower limb isometric strength, quality of life, and neuroplasticity, in terms of plasma BDNF levels, in prefrail older adults.

## 2 Methods and analysis

### 2.1 Design and setting

**FIGURE 1:**
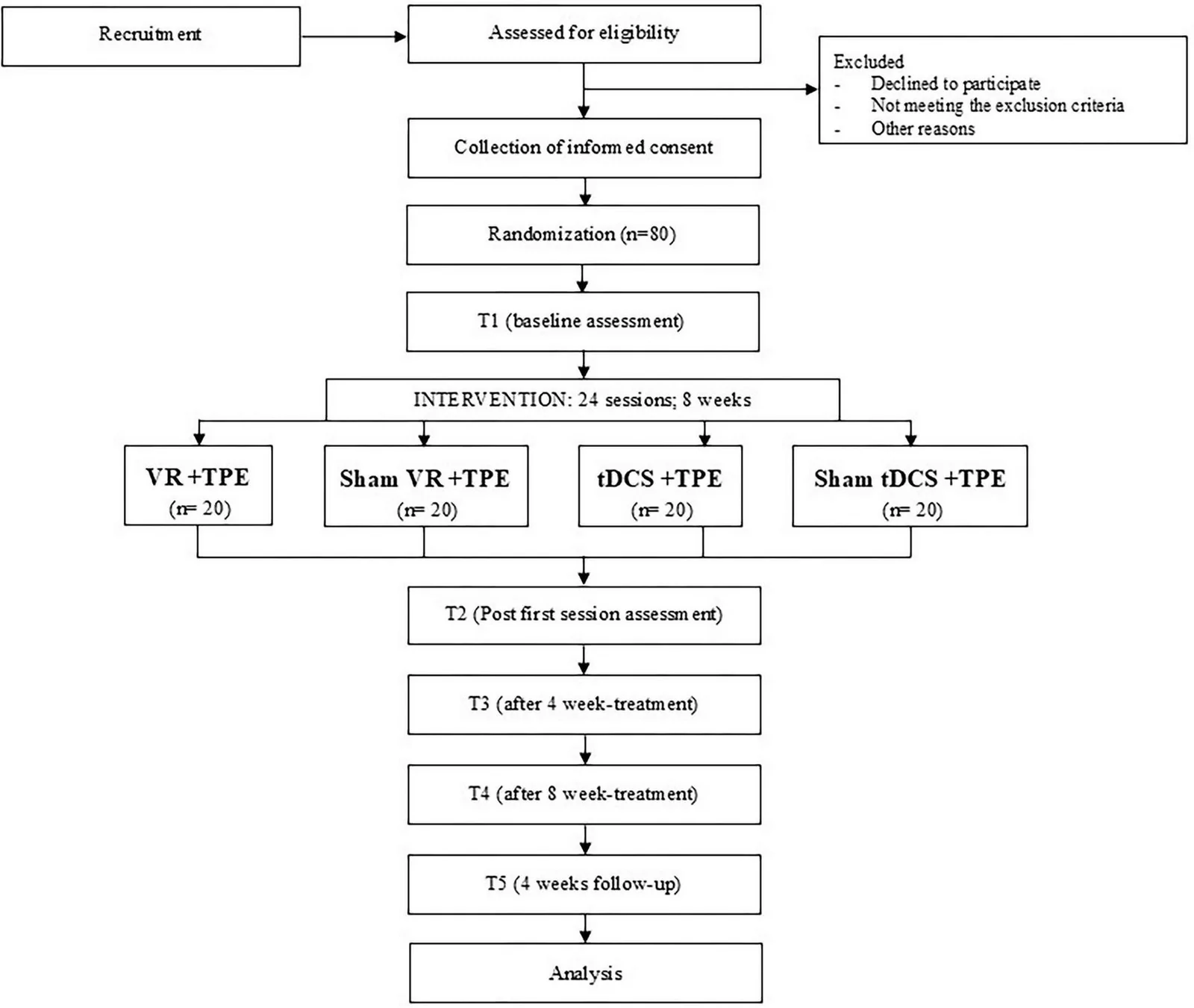
Flowchart of the study design.

This protocol describes a four-arm triple-blinded (participant, outcome assessor, statician) randomised controlled trial that will be conducted in the Faculty of Physiotherapy (University of Valencia). The participants will be randomly allocated into four groups that will be described later: (i) VR + TPE; (ii); sham VR + TPE; (iii) tDCS + TPE and (iv) sham tDCS + TPE. Assessments will be conducted at the following time points: before the first session (T1), immediately after the first session (T2), after 4 weeks of intervention (T3), immediately after the 8-week intervention period (T4) and at 4 week-follow-up (T5). This longitudinal assessment schedule is necessary given the nature of the intervention, which is hypothesized to induce both immediate and experience-dependent neuroplastic changes over time. Early time points (T1–T2) allow the capture of acute neurophysiological and behavioral effects following initial exposure, whereas intermediate and post-intervention assessments (T3–T4) are essential to evaluate progressive training-related adaptations. Finally, the follow-up assessment (T5) enables the examination of the retention and consolidation of treatment-induced effects, providing insight into the durability of potential neuroplastic reorganization. The study design is in accordance with the 2025 Standard Protocol Items: Recommendations for Interventional Trials (SPIRIT) statement (Chan et al., 2025). The flow chart of the study is shown in Figure 1.

### 2.2 Patient and public involvement

Participants have participated and will actively participate in the conduct of this study. During the study design phase, feedback was obtained from prefrail and frail individuals participating in a related study conducted by the same research group, and this input informed the refinement of the intervention procedures and study materials. Furthermore, after finishing the study trial, participants will be provided an individual report via email and provided with a summary of the findings and links to the published manuscripts.

### 2.3 Participants

Participants aged over 65 years will be recruited through different older people’s associations (i.e., La Nau Gran and Asociación Grupo de Mayores de Telefónica). The study will also be advertised on the research group website and social media. The researchers will establish compliance with the selection eligibility criteria described in Table 1. Researchers may terminate a participant’s involvement in the study if changes in the participant’s clinical status result in non-compliance with the established eligibility criteria.

**TABLE 1:** Eligibility criteria.

Eligibility Criteria	
**Inclusion criteria**	**1. Exclusion criteria**
Aged over 65 years	History of stroke within the past 6 months or hospital admission for any reason within the past 3 months
Being prefrail according to Fried criteria	Diseases of the central or peripheral nervous system
Ability to understand instructions (Mini-Mental State Examination > 23 points)	Vestibular system
Signing the written informed consent.	Concomitant diseases Have a neurological pathology, cardiovascular musculoskeletal that contraindicates physical activity Epilepsy or history, medications that lower the seizure threshold Cardiac pacemaker, endocranial and hearing implants History of severe headaches Uncontrolled intracranial or arterial hypertension Heart and/or respiratory failure Implanted medication pump Skin lesions (psoriasis, eczema) Serious head surgeries Completing less than 80% of training sessions

### 2.4 Intervention and comparator

All the interventions will be administered for 24 sessions, 3 days a week, by professional physiotherapists (i.e., more than 5 years of experience). During the intervention, participants will continue their usual pharmacotherapy (which will be registered for subsequent analysis), but other simultaneous physiotherapy treatments will be forbidden. Potential adverse effects arising during the intervention phase or during the follow-up period will be systematically monitored and recorded by the research team. Should an adverse event require clinical attention, the lead investigator will coordinate and guarantee the implementation of the necessary therapeutic measures.

#### 2.4.1 Experimental intervention 1: VR + TPE

The participant will be instructed to walk at a somewhat-hard to hard-pace (i.e., 4–5 on the Borg Scale of rated perceived effort (RPE)) on a treadmill, with a mirror located just in front of him at waist height. He will be asked to watch at a height-adjustable screen (from waist to caudal) where a video of real legs running on a treadmill will be projected for 20 min. To ensure that the participant feels the projected legs as their own, projected legs will be adapted to each subject according to their height and weight. After 20 min of VR + walking, the participant will undergo a multicomponent TPE training for 40 min, including strength, balance, coordination and mobility. The Borg Scale was administered during each session to assess participants’ perceived exertion, including effort, fatigue and breathlessness experienced throughout the intervention in order to monitor and control session-related fatigue and ensure that exertion did not exceed the prescribed threshold. When participants reported an exertion level below “moderate” on the scale, the intensity was progressively increased by introducing greater external loads and/or incorporating unstable surfaces to enhance the overall physical demand. This instrument has demonstrated high reliability (Intraclass Correlation Coefficient, ICC = 0.88 to 0.93) (Mendelsohn et al., 2008).

#### 2.4.2 Placebo Comparator 1: sham VR + TPE

The intervention will be the same as the former group, yet the video projection will consist of landscapes without featuring any type of human or animal movement, in order not to stimulate the motor areas of the brain.

#### 2.4.3 Experimental intervention 2: tDCS + TPE

Anodal tDCS (a-tDCS), will be administered, using a battery-driven direct current stimulator (EPTE^®^ Bipolar System, Ionclinics, Spain). Two saline-soaked sponge electrodes (35 cm^2^) will be secured in place by a neoprene helmet, in accordance with the EEG 10–20 International System, for optimal targeting of the primary motor cortex M1. The anodal electrode will be located on the primary motor cortex (M1), while the cathode will be positioned over the contralateral supraorbital cortex. The treatment parameters will be: stimulation time of 20 min; intensity of 2 mA, current rise-fall time at the beginning and end of stimulation of 30 s. The 20-minute tDCS session will be administered concurrently with treadmill walking. Following the stimulation period, participants will proceed to perform the TPE protocol described above.

#### 2.4.4 Placebo Comparator 2: sham tDCS + TPE

The instrumentation and setting will be the same as in the real tDCS intervention (i.e., tDCS while walking on a treadmill), but the device will be programmed to increase the intensity from 0 to 2 mA progressively during the first 30 s and then cease with a 30-seconds ramp-down, thus providing the participant with the initial itchy sensation on the skin, similar to the real application. In prior studies, this approach has been shown to ensure participant blinding regarding the stimulation type (active vs. sham) (Antal et al., 2017). They will then perform the TPE protocol explained above.

### 2.5 Outcomes

#### 2.5.1 Primary outcome measures

##### 2.5.1.1 Frailty condition

Frailty status will be assessed using the Fried Frailty Phenotype. Participants will be classified as frail if they meet three or more of the following criteria: (i) unintentional weight loss greater than 4.5 kg in the previous year; (ii) self-reported exhaustion; (iii) low level of physical activity; (iv) slowness, determined by gait speed; and (v) muscle weakness, assessed by handgrip strength. Participants meeting one or two criteria will be classified as pre-frail, whereas those meeting none of the criteria will be considered robust. The Fried Frailty Phenotype has demonstrated good predictive validity and reliability for adverse health outcomes in older adults (Fried et al., 2001).

##### 2.5.1.2 Walking speed

The 10-Meter Walk Test will be used to assess walking speed. The participant will be instructed to walk as fast as possible over a total distance of 14 meters, including 2-meter acceleration and 2-meter deceleration phases. Maximum walking speed will be recorded over the intermediate 10-meter distance. The average of three trials (1-minute rest between them) will be calculated for subsequent analysis. The test has demonstrated excellent reliability, with ICC values ranging from 0.89 to 0.99 (Perera et al., 2006).

#### 2.5.2 Secondary outcome measures

##### 2.5.2.1 Neuroplasticity biomarkers

Neuroplasticity biomarkers will be determined by assessing plasma BDNF levels. A nurse will draw blood samples in BD Vacutainer^®^ tubes, with EDTA as an anticoagulant. After being centrifuged at 1500 *g* for 15 min at 4°C, the supernatant (total plasma) will be centrifuged again at 10,000 *g* for 10 min at 4 °C to remove platelets (Rasmussen et al., 2009); then, the resulting supernatant (platelet-free plasma) will be collected in Eppendorf-type tubes, which will be frozen at −80°C until subsequent analysis. For the determination of plasma BDNF levels, the ChemiKine™ BDNF Sandwich kit for ELISA (Millipore, Temecula, CA, USA) will be used according to the manufacturer’s instructions.

##### 2.5.2.2 Heart rate variability

Heart rate variability (HRV) will be continuously recorded using a chest strap device (Polar H10; Polar Electro Oy, Kempele; Finland) at three different time points: (i) 5 min at rest while lying in a supine position at the beginning of each assessment session; (ii) during the 6 mWT to evaluate the effect of the intervention in HRV in the exertional test at each of the time assessment points; (iii) during the first 20 min of the first session of each experimental intervention (i.e., VR/tDCS + walking) to explore acute effects of each intervention. Time-domain HRV indices will include mean RR interval duration (RRi), the standard deviation of normal-to-normal intervals (SDNN), the root mean square of successive differences between adjacent normal RR intervals (rMSSD), and the proportion of consecutive RR intervals differing by more than 50 ms (pNN50). Frequency-domain parameters will encompass low-frequency power (LF; 0.04–0.15 Hz), high-frequency power (HF; 0.15–0.40 Hz), the LF/HF ratio, and total power (TP). For statistical analysis, only the final segment of the recording will be considered, with the initial 2 min excluded to account for the stabilization phase (Pereira et al., 2016).

Data will be exported as.txt files from the Elite HRV application (Elite HRV LLC, USA) and subsequently analyzed using Kubios HRV version 3.5 (Kubios Oy, Finland). The recordings will be carefully reviewed to detect artifacts and ectopic beats (less than 3%), which will be manually corrected and substituted through interpolation based on adjacent RR intervals (Pereira et al., 2016).

##### 2.5.2.3 Muscle voluntary isometric strength

The maximum voluntary isometric strength of the lower limb muscles will be carried out using a load cell (Strength sensor kit, Chronojump Boscosystem^®^, Barcelona) connected to the corresponding software. This load cell has been previously validated and used in geriatric populations (Gaudet and Handrigan, 2020). Specifically, the gluteus medius, quadriceps, and hamstrings strength will be measured. Each measurement will be repeated 3 times (5 s contraction, 30 s rest), with a 2-minute rest between muscle assessments. The maximum and mean strength in the central 3 s of the contraction phase will be recorded from each repetition, then the average of the three repetitions will be used for the subsequent data analysis. To prevent accumulated fatigue from biasing the data, the sequence of muscle group assessments will be randomized across participants. Any repetitions showing a variation greater than 20% will be excluded, and an additional measurement will be subsequently obtained. On the other hand, upper limb strength will be assessed by determining handgrip strength with a hand dynamometer (Jamar™ Hidraulic Hand Dynamometer (Preston, Jackson, Missouri, EE.UU.). Three measurements of the dominant hand will be taken and the mean will be considered for further analysis. Higher values indicate greater muscle strength.

##### 2.5.2.4 Quality of life EQ-5D-5L

Health-related quality of life will be evaluated using the EuroQol five-dimension, five-level questionnaire (EQ-5D-5L). This instrument consists of five domains reflecting key aspects of individual well-being and functional status: mobility, self-care, usual activities, pain/discomfort, and anxiety/depression. EQ-5D-5L index values will be derived using the Crosswalk Index Value Calculator provided by the EuroQol Group (Euroqol, 2023). Scores will range from -0.654 to 1, with values approaching 1 representing a more favorable health condition. The questionnaire has demonstrated excellent reliability, with ICC equal to or greater than 0.75 (Feng et al., 2021).

##### 2.5.2.5 Lower limb muscle power

Lower limb muscle power will be evaluated using the Five Times Sit-to-Stand Test (5STST). This test quantifies the time required for an individual to stand up and sit down five consecutive times from a chair as quickly as possible, without using the upper limbs for support. This test has demonstrated excellent test–retest reliability in older adults, with ICC ranging from 0.89 to 0.99 (Bohannon, 2011).

##### 2.5.2.6 Static and dynamic functionality and gait quality

Postural control and gait quality will be assessed using an inertial sensor integrated into the Android-based FallSkip^®^ system (Biomechanical Institute of Valencia, Spain) (Serra-Añó et al., 2020), following a previously validated protocol developed by our research group (Serra-Añó et al., 2019). The sensor will be positioned at the L4–L5 level, approximating the body’s center of mass. The assessment will consist of a continuous sequence including: (i) quiet standing for 30 s; (ii) walking as fast and as safely as possible over a 3-meter distance toward a chair at the sound of an acoustic signal; (iii) turning and sitting down; (iv) standing up; and (v) returning to the starting point at maximal safe speed. During the static standing phase, medial–lateral and anterior–posterior displacement, as well as the sway area of the center of mass, will be calculated in mm and mm^2^, respectively. Gait analysis will include vertical and medial–lateral range of the center of mass. Transitional movements (i.e., turning, sitting, and standing) will provide measures of: (i) standing-to-sitting time, (ii) standing- up power and (iii) sitting-to-standing time. Additionally, total task duration and reaction time to the auditory stimulus will be recorded.

##### 2.5.2.7 Static and dynamic functionality and gait quality with a dual task

The procedure will replicate the previously described assessment protocol, with the addition of a dual-task condition (i.e., a concurrent cognitive task consisting of serial numerical subtraction from a random three-digit number while walking) (Liao et al., 2021; Zhou et al., 2014).

### 2.6 Randomization (sequence generation, allocation concealment mechanism, implementation) and blinding

Participants will be assigned to the study groups (allocation ratio 1:1:1:1:1:1), through a computer-generated randomization sequence (SPSS statistical software, v29). This procedure will be conducted by an independent researcher who has no direct involvement in the trial, ensuring the neutrality of the allocation process. Group assignment will be concealed using sequentially numbered, opaque, sealed envelopes, which will be opened only after each participant is formally enrolled in the study. In addition, both the evaluator responsible for outcome assessments and the researcher who will perform the statistical analysis will be blinded to group allocation in order to minimize potential bias in data collection and interpretation.

### 2.7 Sample size

Sample size has been calculated *a priori* using a type I error of 5% and a statistical power of 80% using the software GPower 3.1 (Universität Düsseldorf, Germany). Likewise, an effect size that would allow the minimum detectable change reported by Perera et al. (2006) was considered, based on the main variable ‘speed’ of 0.19 m/s, with a standard deviation of 0.29 m/s. Based on these premises, the number of participants required per group is 16. Assuming potential losses of 20%, the necessary sample size will be of 20 participants per group for a total sample of 80 participants.

### 2.8 Data collection, management and confidentiality

Data collection will be conducted by trained physiotherapists assessors following standardized operating procedures to ensure consistency across the five assessment time points (T1–T5). All outcome measures, including functional tests, questionnaire data, and biochemical analyses, will be recorded using pre-specified case report forms and subsequently entered into a secure, password-protected electronic database hosted on institutional servers. To ensure confidentiality, each participant will be assigned a unique identification code to guarantee pseudonymization, and personal identifiers will be stored separately from research data. Data entry will be independently verified by a second researcher to minimize transcription errors, and automated range and consistency checks will be applied to detect implausible values. Access to the final dataset will be restricted to the principal investigator and the statistician, the former being blinded to group allocation. All study documents will be stored in locked cabinets in restricted-access facilities, and electronic data will be retained for the period required by institutional and regulatory policies. Data protection procedures will be reviewed and supervised by the Data Protection Officer, who oversees compliance with applicable data protection regulations. The University of Valencia is fully compliant with the General Data Protection Regulation (GDPR) and national data protection legislation. For any inquiries, suggestions, requests for the exercise of data subject rights, or amicable resolution of data protection-related issues, a dedicated contact address (lopd@uv.es) will be available, without prejudice to the right to lodge a complaint with the competent supervisory authority.

### 2.9 Statistical analysis

Data analysis will be performed using the statistical program SPSS v29. For all studies, sample normality will be analysed using the Shapiro Wilk test, homoscedasticity using Levene’s test, and sphericity for the intra-subject factor using Mauchly’s test. For the comparison between groups, a mixed- design ANOVA will be used, with the between-subjects factor “group” (with the two groups included in each sub-study) and the intra-subject factor “time” including the five assessments. The dependent variables will be those described in the variable description section. The Bonferroni correction will be used for *post-hoc* comparisons. If there is a potential confounding factor that meets the requirements (independence and homogeneity of the lines) to be analysed as a covariate, ANCOVA will be used. The possible heterogeneity of the samples from each pair of groups (in each sub-study) in the baseline will be analysed using an independent samples One-way ANOVA. Differences will be considered statistically significant when the *p*-value is less than 0.05.

### 2.10 Data and trial monitoring and protocol amendments

Given the low-risk nature of the interventions and their non-pharmacological design, a formal Data Monitoring Committee will not be established for this trial. However, an independent senior researcher, not involved in participant recruitment, intervention delivery, or outcome assessment, will be appointed to oversee trial conduct and data integrity. This supervisor will periodically review recruitment procedures, protocol adherence, completeness and accuracy of data entry, and the reporting of adverse events. Any protocol deviations or unexpected safety concerns will be documented and communicated to the interested parties (Ethics Committee, research team and participants).

## 3 Discussion

This protocol presents a randomized controlled trial designed to examine the effectiveness of two distinct non-invasive neuromodulation strategies—visual illusion–based VR and tDCS—combined with a multicomponent TPE programme on functional capacity, neuroplasticity, and quality of life in prefrail older adults. This study addresses a critical gap in the field: although TPE remains the cornerstone intervention for delaying or reversing frailty, many older adults struggle to achieve the intensity needed to elicit multisystemic adaptations due to fatigue, mobility limitations, or reduced functional reserve (World Health Organization, 2010). As a result, their ability to benefit fully from TPE-only approaches is often compromised. Therefore, incorporating this type of non-active intervention alongside TPE may enhance the beneficial effects of exercise itself, without requiring exposure to high levels of fatigue that could reduce adherence to the intervention.

Frailty is increasingly understood as a condition involving not only physical decline, but also alterations in cortical structure, neural connectivity, and sensorimotor integration (Maltais et al., 2020; Tian et al., 2020). These neural changes contribute to slower gait, impaired coordination, reduced motor control, and a decline in executive functions essential for managing complex movements (Carp et al., 2011; Goh, 2011; Koen et al., 2019). Therefore, combining neuromodulation with TPE may enhance the neuroplastic responses required for functional improvement. The two neuromodulation modalities included in this protocol influence brain activity through different mechanisms: VR activates mirror neuron networks involved in motor representation and action understanding (Gandhi et al., 2020; Maggio et al., 2020; Mollà-Casanova et al., 2023; Mollà-Casanova et al., 2024; Moseley, 2007; Nitsche et al., 2003; Peng et al., 2019) whereas tDCS modulates cortical excitability via stimulation on neuronal membrane potential (Manor et al., 2016; Nitsche et al., 2003; Zhou et al., 2015), However, despite growing evidence supporting these techniques in neurological and geriatric conditions, their combined effect with TPE has not yet been investigated in prefrail adults—an important population given the reversible nature of frailty (Abellan van Kan et al., 2008).

If this study demonstrates that VR or tDCS combined with TPE produce greater or comparable improvements relative to TPE alone, the results may suggest the potential clinical value of these approaches as adjunctive interventions. A further strength of this trial is the inclusion of neuroplasticity biomarkers, since it is of great interest to understand the molecular mechanisms involved in the neuroplasticity induced by these types of therapies. There are numerous proteins considered neurotrophic with different functions in the nervous system, such as nerve growth factor (NGF), insulin-like growth factor (IGF-I), neurotrophin 3 (NT-3) and 4/5 (NT 4/5), and brain-derived neurotrophic factor (BDNF) (Noble et al., 2011). The most abundant is BDNF, which stimulates the development and differentiation of new neurons, the survival of existing ones, synapses and neuronal plasticity, promoting long-term potentiation (Miranda et al., 2019). Lower BDNF levels have been associated with frailty and poorer cognitive status (Inglés et al., 2017), and previous research has shown that exercise (Gómez-Pinilla et al., 2002) and tDCS (Fritsch et al., 2010) can independently elevate BDNF. Determining whether their combination yields additive or synergistic effects will provide valuable insights into the neural mechanisms underpinning functional improvements in prefrailty. This biological perspective is especially relevant as interventions that enhance neuroplasticity may also improve cognitive and dual-task performance, both of which are impaired early in the frailty spectrum.

The methodological design of this study—including a four-arm structure, blinding of participants, assessors, and statisticians, and the use of sham controls specific to each technique—ensures a high degree of internal validity. The comprehensive assessment strategy, including questionnaires, objective biomechanical instruments, functional tests and physiological measurements will allow a multidimensional understanding of how neuromodulation influences motor and systemic functions. The addition of dual-task assessments further strengthens the protocol by evaluating cognitive–motor integration, a domain highly relevant to fall risk and functional independence.

Regarding the feasibility of the study, participant commitment will be emphasized before enrolment and only individuals willing to attend the complete intervention programme will be included. Previous studies conducted by our research group in prefrail and frail older adults have consistently demonstrated high adherence and motivation, likely because participants perceive these interventions as beneficial for preserving functionality, independence and health. Adherence rates, fatigue, adverse events and attrition will be systematically recorded throughout the study to further assess feasibility.

Overall, this study aims to provide evidence regarding the potential of neuromodulation techniques to enhance or complement the effects of a standardized TPE programme in prefrail older adults. If the hypotheses are confirmed, the findings could support the development of simple and generalizable therapeutic protocols that consider both the individual’s physical capabilities and their neurophysiological responsiveness. This could ultimately contribute to preventing the progression toward frailty and reducing the associated health and social burdens.

## 4 Ethics and dissemination

The study has already been approved by the Ethics Committee of Research in Humans of the Ethics Commission in Experimental Research of University of Valencia (2025-FIS-4007899). The study will be conducted in accordance with the ethical principles outlined in the Declaration of Helsinki. Before enrolment, all participants will receive detailed information regarding the objectives, procedures, benefits and potential risks of the study. They will be explicitly advised of their right to withdraw from the study at any point without any adverse consequences. Every participant will voluntarily sign the informed consent form (Supplementary material: informed consent) before their formal inclusion in the study.
